# QTc Interval Predicts Disturbed Circadian Blood Pressure Variation

**DOI:** 10.1515/med-2020-0021

**Published:** 2020-03-06

**Authors:** Liyuan Yan, Jianling Jin, Shili Jiang, Wei Zhu, Meiwen Gao, Xin Zhao, Jiamin Yuan

**Affiliations:** 1 Department of Cardiology, the First Affiliated Hospital of Soochow University, No.188, Shizi Road, Suzhou, 215006, Jiangsu, China; 2 Department of Electrocardiography, the First Affiliated Hospital of Soochow University, Suzhou, Jiangsu, 215006, China

**Keywords:** Ambulatory blood pressure monitoring, Circadian blood pressure rhythm, Electrocardiography, Hypertension

## Abstract

**Background:**

The relationship between electrocardiographic evaluation and circadian blood pressure (BP) variation in young and middle-aged hypertensive patients remains unknown.

**Methods:**

A total of 171 hypertensive patients were included in the study. First, patients were divided into a young and middle-aged group and an elderly group. The two groups were then separately classified into three subgroups on the basis of circadian variation of BP as dippers, non-dippers and reverse-dippers. The electrocardiographic evaluation was calculated from 12-lead electrocardiography (ECG).

**Results:**

QTc intervals were shortest in the dippers and longest in the reverse-dippers in the young and middle-aged group (QTc dipper: 416.53±18.37ms; non-dipper: 438.30±29.71ms; reverse-dipper: 444.93±25.47ms; for dipper vs non-dipper, and dipper vs reverse-dipper P<0.05). QTc interval was found to be an independent risk factor for the non-dipper BP pattern (Odds ratio 1.049; 95% CI 1.01-1.089; P=0.012) and reverse-dipper BP pattern (Odds ratio 1.051; 95% CI 1.007-1.098; P=0.023) in young and middle-aged hypertensive patients. No significant differences in other ECG parameters were found among the three subgroups in the young and middle-aged group.

**Conclusion:**

Our study suggested that QTc interval might serve as a risk factor for non-dipper BP pattern and reverse-dipper BP pattern in young and middle-aged hypertensive patients.

## 1 Introduction

Hypertension, ranking among the most prevalent chronic diseases, not only causes health loss by itself, but also acts as an independent risk factor for many other diseases, such as stroke, heart failure, renal failure and coronary artery disease [1]. To prevent target organ damage and cardiovascular events, the latest hypertension guidelines recommend early, strict and all-day blood pressure control [2, 3]. Therefore, ambulatory blood pressure monitoring (ABPM) is increasingly used in clinical practice to analyze circadian BP variation, to detect nocturnal hypertension and to evaluate the efficacy of antihypertensive drugs [4]. Circadian BP variation is quantified through the diurnal/nocturnal BP ratio. Based on it, BP rhythms can be divided into four categories, which are known as extreme-dipper, dipper, non-dipper and reverse-dipper [5]. Studies have shown that blunted nocturnal blood pressure dipping is related to damage of end organs and to cardiovascular mortality [6, 7].

There have been several studies investigating the association between electrocardiographic evaluation [eg: heart rate , frontal QRS-T angle, QTc interval, Tpeak to Tend interval and left ventricular hypertrophy (LVH)] and the circadian variation of BP [8, 9, 10]. However, the effect of circadian variation of BP on electrocardiographic evaluation remains controversial. What’s more, to the best of our knowledge, there is no study investigating the relationship between electrocardiographic evaluation and BP reverse dipping status in young and middle-aged patients with essential hypertension. Therefore, this study is designed to further investigate the relationship between electrocardiographic evaluation and circadian BP variation in young and middle-aged hypertensive patients.

## 2 Methods

### 2.1 Study population

This single-center study retrospectively included hypertensive patients admitted to the Cardiology department at the first Affiliated Hospital of Soochow University who simultaneously underwent ECG and ABPM from January 2017 to January 2019. Hypertension was defined as previously diagnosed hypertension, currently using antihypertensive drugs, office-measured systolic BP (SBP)/diastolic BP (DBP)≥140/90 mmHg, 24h ABPM SBP/DBP≥130/80 mmHg, daytime (or awake) ABPM SBP/DBP≥135/85 mmHg or nighttime (or sleep) ABPM SBP/DBP≥120/70 mmHg[3]. Patients with age < 60 years old were considered as young and middle-aged patients, while those with age ≥ 60 years old were defined as elderly patients [11, 12]. Body mass index was calculated as weight in kilograms divided by the square of the height in meters. The patients were excluded if they 1) had incomplete medical records; 2) were <18 or >90 years old; 3) were taking drugs that may affect QTc interval duration in addition to antihypertensive drugs; 4) also had secondary hypertension, atrial fibrillation, atrial flutter, bundle branch block, II/III-degree atrioventricular block, pre-excitation, paced rhythm, diabetes mellitus, chronic liver disease, chronic renal disease, acute coronary syndrome, moderate or severe valvular disease, chronic obstructive pulmonary disease, thyroid function disorders, electrolyte imbalance, heart failure, obstructive sleep apnea syndrome or malignant tumor.

There were only two extreme-dippers meeting the above inclusion and exclusion criteria. Due to the limited numbers, the two extreme-dippers were not included in our study. In the end, a total of 171 patients were included in the study. They were first divided into two groups, the young and middle-aged group and the elderly group. Then, the above two groups were separately classified into three subgroups on the basis of circadian variation of BP, as dipper, non-dipper and reverse-dipper.

### 2.2 ABPM recordings

ABPM was performed to record the circadian variation of blood pressure in all included patients by an ABPM 6100 device (Welch Allyn Corp., NY, USA). The cuff was placed on the right arm of patients. Patients were asked to keep their daily routine and stay calm when feeling the inflation of cuff. During the daytime (8:00AM to 11:00PM), BP was measured every 15 minutes. During the nighttime (11:00PM to 8:00AM), BP was measured every 30 minutes. Only recordings with more than 70% of valid BP measurements were included in the final analysis. The means of SBP and DBP were calculated at 24 h, awake and sleep. The nocturnal dip rate was calculated as follows: (%) 100× [1− (average night SBP/average awake SBP)]. Then, the circadian variation of blood pressure status was defined as: extreme-dipper (nocturnal dip rate ≥20%), dipper (10%≤ nocturnal dip rate <20%), non-dipper (0≤nocturnal dip rate<10%) and reverse-dipper (nocturnal dip rate <0) [5].

### 2.3 Electrocardiographic evaluation

All patients included in the study underwent a resting 12-lead ECG (Nalong Corp., Xiamen, China) with a 25mm/s paper speed and 10 mm/mV height. Heart rate and frontal QRS-T angle were obtained directly from ECG reports. The QT intervals were measured using a software program (Nalong Corp., Xiamen, China). Then we recorded the longest QT interval from all limb and precordial leads and QTc interval was calculated using Bazett’s formula[13]. QTc interval >450ms for men and >460ms for women was considered prolonged. Tpeak to Tend (TpTe) interval was measured manually from the peak to the end of the T-wave at the lead V5 when possible [14]. Left ventricular hypertrophy was defined according to Cornell criteria (RaVL+SV3 >28mm for men and >20mm for women) or Sokolow-Lyon (SV1+RV5,6 ≥35mm) [15, 16].

### 2.4 Laboratory tests

Baseline demographic, clinical and laboratory data were collected from the hospital medical records and case collection and scientific research system for clinical cardiology (CCSSSCC). Peripheral blood samples were drawn from patients when they were in a fasting state. Serum levels of alanine aminotransferase (ALT), aspartate aminotransferase (AST) and other biochemical parameters were determined at the Biochemistry Department in the first Affiliated Hospital of Soochow University.

### 2.5 Statistical analysis

Statistical analysis was performed using IBM SPSS for Windows version 25.0 (IBM Corp., Armonk, NY, USA). Continuous variables were tested for a normal distribution using the Shapiro-Wilk test. Continuous variables with a normal distribution were defined as mean±standard deviation (SD), while continuous variables without a normal distribution were defined as median (interquartile range, IQR). Continuous variables were compared using the one way analysis of variance (ANOVA) or the Kruskal-Wallis test, if appropriate. If the results of ANOVA were statistically significant, post-hoc Bonferroni test was performed. If the results of Kruskal-Wallis test were statistically significant, the results of pairwise comparison were corrected by Bonferroni correction. Categorical variables, defined as percentages, were compared using the χ2-test or the Fisher’s exact test, if appropriate. To assess the correlation between continuous variables, Pearson’s correlation coefficients were calculated. To explore the relationship between relevant variables and circadian blood pressure rhythm, multivariate logistic regression analyses were applied. For all analyses, values of P less than 0.05 were regarded to be statistically significant.

**Ethical issues**: Our study was in accordance with the principles outlined in the Declaration of Helsinki and approved by the ethics committee of Soochow University.

**Informed consent**: Informed consent has been obtained from all individuals included in this study.

## 3 Results

In the end, a total of 171 patients were included in our study. The baseline demographic and clinical characteristics are shown in Table 1. In the young and middle-aged group, there were significant differences with respect to age, use of diuretics, sleep SBP and sleep DBP among dippers, non-dippers and reverse-dippers [age: 36 (29~48) vs 49 (39~53) vs 47 (51~54) years old in dippers, non-dippers and reverse-dippers, respectively, P=0.048; use of diuretics: 0 (0%) vs 12 (30.0%) vs 5 (35.7%) in dippers, non-dippers and reverse-dippers, respectively, P=0.026; sleep SBP:118 (108~124) vs 118 (108~138) vs 141 (122~167) mmHg in dippers, non-dippers and reverse-dippers, respectively, P=0.006; sleep DBP: 68 (60~81) vs 72 (64~83) vs 86 (70~93) mmHg in dippers, non-dippers and reverse-dippers, respectively, P=0.044]. Other characteristics were similar among the three subgroups in young and middle-aged patients. In the elderly group, there were only sleep SBP and sleep DBP showing significant differences among the three subgroups [sleep SBP: 118 (104~124) vs 123 (112~131) vs 128 (118~142) mmHg in dippers, non-dippers and reverse-dippers, respectively, P=0.015; sleep DBP: 59 (56~64) vs 66 (60~71) vs 69 (63~76) mmHg in dippers, non-dippers and reverse-dippers, respectively, P=0.016].

**Table 1:** Demographic and clinical characteristics of included patients.

	Young and middle-aged patients (n=69)				Elderly patients (n=102)			
	Dipper (n=15)	Non-dipper (n=40)	Reverse-dipper (n=14)	P	Dipper (n=10)	Non-dipper (n=48)	Reverse-dipper (n=44)	P
Age, years	36 (29~48)	49 (39~53)	47 (51~54)	**0.048**	68 (63~70)	68 (62~71)	66 (64~71)	0.960
Sex, male, n (%)	10 (66.7)	21 (52.5)	7 (50)	0.586	2 (20.0)	17 (35.4)	17 (38.6)	0.628
BMI, kg/m^2^	24.53±3.36	24.40±3.72	26.15±3.22	0.278	23.62±2.82	24.47±3.34	25.05±3.05	0.392
Smoking, n (%)	4 (26.7)	9 (22.5)	2 (14.3)	0.729	1 (10.0)	1 (2.1)	5 (11.4)	0.126
Drinking, n (%)	4 (26.7)	4 (10.0)	2 (14.3)	0.320	1 (10.0)	1 (2.1)	2 (4.5)	0.303
Medications, n (%)								
ACEI/ARBs	7 (46.7)	22 (55.0)	10 (71.4)	0.387	7 (70.0)	33 (68.8)	31 (70.5)	1.000
β-Blockers	4 (26.7)	23 (57.5)	7 (50.0)	0.125	5 (50.0)	20 (41.7)	22 (50.0)	0.737
CCBs	9 (60.0)	26 (65.0)	11 (78.6)	0.567	8 (80.0)	31 (64.6)	23 (52.3)	0.219
Diuretics	0 (0)	12 (30.0)	5 (35.7)	**0.026**^^a^^b^^	3 (30.0)	16 (33.3)	9 (20.5)	0.375
24h SBP, mmHg	132 (122~136)	123 (116~143)	138 (120~154)	0.227	129 (116~138)	128 (115~134)	123 (113~136)	0.783
24h DBP, mmHg	79 (72~92)	77 (69~85)	80 (68~93)	0.779	69 (62~74)	68 (62~75)	68 (61~75)	0.938
Awake SBP, mmHg	135 (128~140)	126 (119~144)	135 (119~152)	0.178	132 (120~143)	130 (117~136)	122 (112~135)	0.216
Awake DBP, mmHg	85 (76~95)	79 (70~88)	77 (68~91)	0.435	72 (65~78)	68 (62~76)	68 (60~75)	0.360
Sleep SBP, mmHg	118 (108~124)	118 (108~138)	141 (122~167)	**0.006**^^b^^c^^	118 (104~124)	123 (112~131)	128 (118~142)	**0.015**
Sleep DBP, mmHg	68 (60~81)	72 (64~83)	86 (70~93)	**0.044**^^b^^	59 (56~64)	66 (60~71)	69 (63~76)	**0.016**^^b^^

Normally distributed data are presented as means ± standard deviation (SD), skewed data are presented as the median (interquartile range), and categorical data are presented as a number (percentage). Abbreviations: ACEI, angiotensin-converting enzyme inhibitor; ARB, angiotensin receptor blocker; BMI, body mass index; CCB, calcium channel blocker; DBP, diastolic blood pressure; SBP, systolic blood pressure. Values in bold indicate statistical significance (p < 0.05).

a significant difference between dipper and non-dipper.

b significant difference between dipper and reverse-dipper.

c significant difference between non-dipper and reverse-dipper.

**Figure 1:**
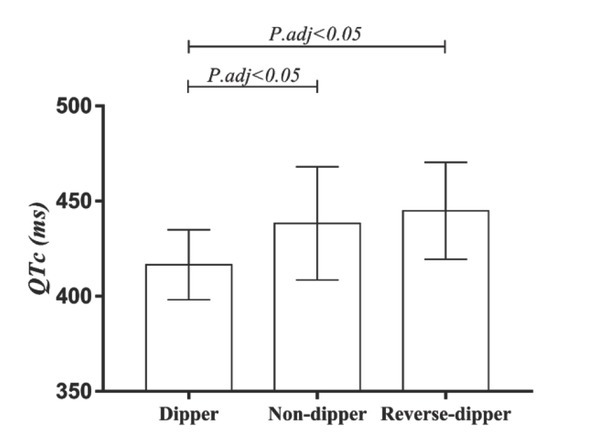
QTc interval given separately for dippers, non-dippers and reverse-dippers in young and middle-aged patients.

The laboratory and electrocardiographic variables of the study population are shown in the Table 2. There were no significant differences in serum levels of creatinine, ALT, AST, potassium (K) and sodium (Na) among the three subgroups in the young and middle-aged group or the elderly group. Heart rate, TpTe, frontal QRS-T angle and the number of patients with electrocardiographic LVH were similar among the dippers, non-dippers and reverse-dippers in the young and middle-aged group. QTc interval and the number of patients with prolonged QTc interval were significantly different among the three subgroups in young and middle-aged hypertensive patients [QTc interval: 416.53±18.37 vs 438.30±29.71 vs 444.93±25.47ms in dippers, non-dippers and reverse-dippers, respectively, P=0.011 (Figure 1); the number of patients with prolonged QTc interval was: 0(0%) vs 12(30.0%) vs 4(28.6%) in dippers, non-dippers and reverse-dippers, respectively, P=0.035]. However, in the elderly group, both QTc interval and the number of patients with prolonged QTc interval were similar among the three subgroups, as were TpTe, frontal QRS-T angle and the number of patients with electrocardiographic LVH. Only heart rate was significantly different among dippers, non-dippers and reverse-dippers in elderly patients with essential hypertension (heart rate:69.90±8.81 vs 71.88±11.16 vs 66.07±9.32 beats per minute in dippers, non-dippers and reverse-dippers, respectively, P=0.027).

**Table 2:** Laboratory and electrocardiographic variables of the young and middle-aged and elderly patients given separately for dippers, non-dippers and reverse-dippers.

	Young and middle-aged patients (n=69)				Elderly patients (n=102)			
	Dipper (n=15)	Non dipper (n=40)	Reverse dipper (n=14)	P	Dipper (n=10)	Non dipper (n=48)	Reverse dipper (n=44)	P
Laboratory variables								
Creatinine, umol/L	70 (55~76)	66 (53~77)	66 (52~85)	0.830	62 (57~68)	60 (53~72)	64 (54~80)	0.803
ALT, U/L	20 (17~43)	21 (13~30)	24 (16~41)	0.465	22 (10~27)	18 (14~24)	19 (14~28)	0.790
AST, U/L	17 (13~26)	19 (17~23)	18 (15~21)	0.734	22 (16~24)	20 (17~25)	21 (17~26)	0.759
K, mmol/L	4.02±0.41	3.87±0.35	3.73±0.48	0.159	3.73(3.53~4.01)	3.90(3.68~4.10)	3.86(3.61~4.11)	0.452
Na, mmol/L	141 (139~143)	140.7 (140~143)	140 (140~142)	0.735	141 (140~143)	142 (140~143)	142 (140~143)	0.652
ECG variables								
Heart rate, beats /min	76.07±17.80	76.30±12.74	76.50±11.66	0.996	69.90±8.81	71.88±11.16	66.07±9.32	**0.027**^^c^^
QTc, ms	416.53±18.37	438.30±29.71	444.93±25.47	**0.011**^^a^^b^^	438.80±19.46	443.29±25.37	434.82±25.79	0.274
Prolonged QTc, n (%)	0 (0.0)	12 (30.0)	4 (28.6)	**0.035**^^a^^b^^	2 (20.0)	14 (29.2)	10 (22.7)	0.754
TpTe, ms	89.40±13.90	92.33±15.91	93.86±21.80	0.764	86.30±26.10	87.40±22.54	95.82±22.39	0.172
Frontal QRS-T angle,°	10 (4~21)	9 (4~17)	11 (4~45)	0.589	18 (2~76)	18 (10~35)	17 (8~28)	0.869
ECG LVH, n (%)	3 (20.0)	6 (15.0)	1 (7.1)	0.583	1 (10.0)	10 (20.8)	7 (15.9)	0.745

Normally distributed data are presented as means ± standard deviation (SD), skewed data are presented as the median (interquartile range), and categorical data are presented as a number (percentage). Abbreviations: ALT, alanine aminotransferase; AST, aspartate aminotransferase; ECG LVH, electrocardiographic left ventricular hypertrophy; K, Potassium; Na, Sodium; TpTe, Tpeak to Tend. Values in bold indicate statistical significance (p < 0.05).

c significant difference between non-dipper and reverse-dipper.

a significant difference between dipper and non-dipper.

b significant difference between dipper and reverse-dipper.

Age, QTc interval, TpTe, frontal QRS-T angle, K and creatinine were included in the multivariate logistic regression analysis. Age and QTc interval were found to be significantly different when comparing reverse-dipper BP pattern with dipper pattern or non-dipper BP pattern with dipper pattern (Table 3). QTc interval was found to be an independent risk factor for non-dipper BP pattern (Odds ratio 1.049; 95% CI 1.01-1.089; P=0.012) and reverse-dipper BP pattern (Odds ratio 1.051; 95% CI 1.007-1.098; P=0.023) in young and middle-aged hypertensive patients. Age was also found to be an independent risk factor for non-dipper BP pattern (Odds ratio 1.092; 95% CI 1.012-1.179; P=0.023) and reverse-dipper BP pattern (Odds ratio 1.116; 95% CI 1.013-1.228; P=0.026).

**Table 3:** Multivariate Logistic Regression Analysis for Circadian Blood Pressure Patterns

Variables	Non-dipper versus Dipper		Reverse-dipper versus Dipper		Reverse-dipper versus Non-dipper	
	OR (95% CI)	P	OR (95% CI)	P	OR (95% CI)	P
Age	1.092 (1.012-1.179)	**0.023**	1.116 (1.013-1.228)	**0.026**	1.021 (0.949-1.099)	0.578
QTc	1.049 (1.01-1.089)	**0.012**	1.051 (1.007-1.098)	**0.023**	1.002 (0.975-1.03)	0.869
TpTe	1.02 (0.966-1.077)	0.479	1.026 (0.967-1.089)	0.394	1.006 (0.972-1.041)	0.73
Frontal QRS-T angle	0.992 (0.961-1.024)	0.615	0.996 (0.963-1.03)	0.809	1.004 (0.987-1.021)	0.649
K	1.418 (0.204-9.849)	0.724	0.49 (0.042-5.682)	0.569	0.346 (0.052-2.314)	0.274
Creatinine	1 (0.958-1.044)	0.994	1.027 (0.977-1.08)	0.293	1.027 (0.993-1.063)	0.125

Abbreviations: CI, confidence interval; K, Potassium; OR, odds ratio; TpTe, Tpeak to Tend. Values in bold indicate statistical significance (p < 0.05).

**Figure 2:**
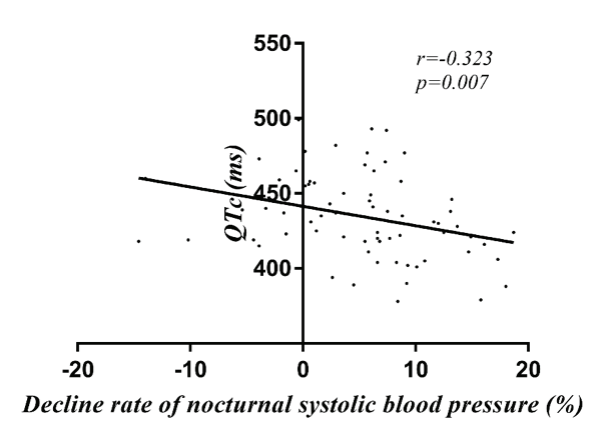
The relationship between QTc and decline rate of nocturnal SBP in young and middle-aged hypertensive patients.

To investigate whether there were associations between age or indices of ABPM and QTc in the young and middle-aged patients, Pearson’s correlation coefficients were calculated (Table 4). QTc interval duration was negatively correlated with decline rate of nocturnal SBP (r=-0.323, P=0.007) in young and middle-aged patients with essential hypertension (Figure 2). However, there was no significant correlation between other parameters and QTc.

**Table 4:** Correlation analysis between age or ambulatory blood pressure recordings and QTc interval in young and middle-aged patients.

	QTc	
	r	p
Age	0.039	0.751
24 h SBP	0.071	0.563
24 h DBP	0.039	0.749
Awake SBP	0.031	0.798
Awake DBP	-0.005	0.968
Sleep SBP	0.173	0.154
Sleep DBP	0.165	0.175
Dipping	-0.323	**0.007**

Abbreviations: DBP, diastolic blood pressure; SBP, systolic blood pressure. Values in bold indicate statistical significance (p < 0.05).

## 4 Discussion

ABPM, which records the variation of blood pressure in 24h, has attracted more and more attention in recent years. Due to its higher reproducibility than office-measured blood pressure, ABPM has been proposed as a clinical tool to confirm office-measured hypertension diagnosis in several international guidelines [2, 3]. Based on the circadian variation of blood pressure, individuals can be divided into four types, as extreme-dipper, dipper, non-dipper and reverse-dipper [5]. Among them, dipper is considered to be a normal status. A reduced nocturnal decline rate or even increase rate in nighttime blood pressure is correlated with end organ damage and adverse cardiovascular events [6, 7, 17].

QTc is one of the most widely used ECG parameters to assess ventricular repolarization. Previous studies have revealed that abnormal prolongation of QTc interval was correlated to ventricular arrhythmia and sudden cardiac death[18, 19, 20]. For every 10ms increase in QTc interval, the risk of cardiac events increases by about 6% [21]. With the increase of 60ms in QTc interval compared to the baseline value, the TdP risk increases [22]. There have been several studies investigating the relationship between QTc interval and the nocturnal drop of blood pressure [8, 9, 10]. Passino et al demonstrated that non-dippers had longer QTc intervals than dippers in hypertensive patients. In addition, they found that circadian rhythmicity of QTc interval was affected by circadian variation of blood pressure [10]. However, Karaagac et al demonstrated that QTc interval was similar between dippers and non-dippers in patients with metabolic syndrome[8]. Moreover, there was no significant difference among dippers, non-dippers and reverse-dippers in patients with prehypertension in another study [9]. Based on the previous studies, the relationship between QTc and circadian variation of blood pressure remains controversial. To the best of our knowledge, there have been no studies assessing the relationship between QTc and reverse dipping status in young and middle-aged patients with essential hypertension.

In our study, the main finding was that QTc interval was significantly longer in non-dippers and reverse-dippers than in dippers in young and middle-aged hypertensive patients. However, QTc was similar among dippers, non-dippers and reverse-dippers in elderly patients with essential hypertension. What’s more, QTc interval duration was negatively correlated with the decline rate of nocturnal SBP in young and middle-aged hypertensive patients. Last but not least, QTc interval and age were found to be independent risk factors for non-dipper BP pattern and reverse-dipper BP pattern in young and middle-aged hypertensive patients.

The relationship between QTc interval and circadian BP variation might be explained by LVH and impaired autonomic nervous system functions. Loss of normal nocturnal BP drop is correlated to left ventricular hypertrophy. Ivanovic et al demonstrated that LVH was most prevalent among reverse-dippers compared to dippers, non-dippers, extreme-dippers [23]. Hypertrophic myocardium may lead to prolongation of QTc interval by altering ventricular repolarization and prolonging action potential duration [10]. Impairment of autonomic nervous system function plays an important role in BP variation [24]. Non-dippers exhibit increasing sympathetic activity and decreasing vagal activity. The reverse dipping status is related to even greater sympathetic activation than non-dipping status [25]. QTc interval is also related to the withdrawal of vagal drive and sympathetic overactivity. Therefore, autonomic nervous system dysfunction may contribute to QTc prolongation in young and middle-aged non-dippers and reverse-dippers. Use of diuretics may result in hypokalemia, which can lead to prolongation of QTc interval [26]. In our study, non-dippers and reverse-dippers used more diuretics than dippers in the young and middle-aged group. However, the serum level of potassium was similar among the three subgroups in young and middle-aged patients. Therefore, use of diuretics may be excluded from reasons for prolongation of QTc in non-dippers and reverse-dippers in young and middle-aged hypertensive patients.

In our study, the correlation between QTc prolongation and blunted nocturnal blood pressure drop was observed in young and middle-aged patients but not in elderly patients. Older age itself is a risk factor for QTc interval prolongation [27]. Elderly people tend to have a longer QTc interval than young and middle-aged people. In addition, elderly patients may have longer histories of hypertension than young and middle-aged patients, resulting in greater LVH which is related to the prolonging of QTc interval. The findings of our study may help to explain the conflicting results of previous studies.

Tanriverdi et al demonstrated that frontal QRS-T angle was smaller in patients with dipper hypertension than in patients with non-dipper hypertension [28]. On the contrary, there was no significant difference among dippers, non-dippers and reverse-dippers in both the young and middle-aged group and the elderly group with respect to QRS-T angle in our study. The inclusion and exclusion criteria in Tanriverdi’s study were quite similar to the criteria in our study. However, their study focused on patients in Turkey, while our study included Chinese patients with essential hypertension.

In our study, heart rate was significantly different among dippers, non-dippers and reverse-dippers in elderly patients with essential hypertension. Moreover, heart rate was lowest in revere-dippers and highest in non-dippers in elderly hypertensive patients, which was inconsistent with previous studies [9, 28]. The reverse dipping status is related to the greatest sympathetic activation, which may result in higher heart rate [25]. However, in our study, reverse-dippers had the lowest heart rate among the three subgroups in elderly hypertensive patients. Therefore, other mechanisms may be involved in the effect of BP variation on heart rate. More studies are needed to further investigate the correlation between heart rate and circadian BP variation.

Recently, the Monitorización Ambulatoria para Predicción de Eventos Cardiovasculares (MAPEC) study found that the reduction of sleep time SBP and correction of blunted night blood pressure drop through a night time antihypertensive treatment strategy can most efficiently lower the risks of stroke and cardiovascular diseases [29, 30]. In our study, only young and middle-aged patients with dipper hypertension exhibited a shorter QTc interval than non-dippers and reverse dippers. The findings of our study may suggest use of ABPM and correction of blunted nocturnal blood pressure drop at an early stage.

## 5 Conclusion

Our study suggests that there is a correlation between QTc interval and the circadian variation of blood pressure in young and middle-aged hypertensive patients but not in the elderly. We found that QTc interval is a risk factor for non-dipper and reverse-dipper status in young and middle-aged hypertensive patients. What’s more, QTc interval is negatively correlated with decline rate of nocturnal SBP. The finding of our study may help to explain the conflicting results of previous studies.
